# Risk factors of hospitalization and readmission of patients with COPD in Hong Kong population: Analysis of hospital admission records

**DOI:** 10.1186/1472-6963-11-186

**Published:** 2011-08-10

**Authors:** Frank WK Chan, Fiona YY Wong, Carrie HK Yam, Wai-ling Cheung, Eliza LY Wong, Michael CM Leung, William B Goggins, Eng-kiong Yeoh

**Affiliations:** 1 School of Public Health and Primary Care, The Chinese University of Hong Kong, Prince of Wales Hospital, Shatin, N.T., Hong Kong

**Keywords:** COPD, Hospitalization, Unplanned Readmission, Hong Kong

## Abstract

**Background:**

Chronic Obstructive Pulmonary Disease (COPD) accounts for around 4% of all public hospital annual admissions in Hong Kong. By year 2020, COPD will be ranked fifth among the conditions with the highest burden to the society. This study identifies admission and unplanned readmission of COPD patients, factors affecting unplanned readmission, and estimates its cost burden on the public healthcare system in Hong Kong.

**Methods:**

This is a retrospective study analyzing COPD admissions to all public hospitals in Hong Kong. All admission episodes to acute medical wards with the principal diagnosis of COPD (ICD-9:490-492, 494-496) from January 2006 to December 2007 were captured. Unplanned readmission was defined as an admission which followed a previous admission within 30 days.

**Results:**

In 2006 and 2007, 65497 (8.0%) of episodes from medical wards were identified as COPD admissions, and among these, 15882 (24.2%) were unplanned readmissions. The mean age of COPD patients was 76.81 ± 9.59 years and 77% were male. Unplanned readmission was significantly associated with male gender, receiving public assistance and living in nursing homes while no association was found with the Charlson comorbidity index. Patients who were readmitted unplanned had a significant longer acute length of stay (β = 0.3894, P < 0.001) after adjustment for other covariates.

**Conclusions:**

Unplanned readmission of COPD patients has a huge impact on the public healthcare system. A systematic approach in programme provision and a good discharge planning process targeting on COPD patients who are at high risk of unplanned readmission are essential.

## Background

Chronic Obstructive Pulmonary Disease (COPD) is described as a group of lung diseases characterized by airflow limitation [1]. COPD is a leading cause of morbidity and mortality worldwide and it remains a major public health problem. It has been estimated that by the year 2020, COPD will be ranked third among the most important causes of death and fifth among the conditions with a high burden to society for both sexes worldwide [2]. In Hong Kong, its prevalence rate has been reported to be 10% among the older population [3].

Acute exacerbation of COPD is a common cause of hospital admission and readmission. The COPD admission rate in the UK doubled between 1991 and 2000 and by 2000 accounted for 1% of all hospital admissions [4]. COPD is also the greatest burden on the health care system [1,5]. In the European Union, the total direct costs of respiratory disease are estimated to be about 6% of the total health care budget, with COPD accounting for 56% (USD 53.9 billion) of the cost spent on respiratory disease [1]. In the US, the direct cost of COPD was US$18 billion in 2002 [1].

Despite the fact that COPD is a common and important condition, studies focusing on risk factors affecting admission or readmission are limited. Unplanned readmission can be related to premature discharge, frequent admissions in the previous year, lack of connectivity of care between hospital and the community and, poor social service support [6]. Clinical factors like relapse of original condition, development of a new problem, complications of the initial illness, as well as the need for terminal care are also possible reasons for unplanned readmission [7]. Patient factors such as poor self-management, carer support and problems with medication can also lead to unplanned readmission [6,7]. Unplanned readmission also has important health policy implications [8]. To a certain extent, it reflects the quality of care [9]. A high unplanned readmission rate may indicate poor clinical management in hospital which will increase the burden on the healthcare system in long term.

Previous studies have been mainly prospective cohorts with the sample sizes were fewer than a thousand [8]. In order to understand the factors related to COPD admission and readmission and to enhance the formation of health policies and their implementations, in this study we analyzed the potential factors using a unique dataset obtained from the Hospital Authority (HA) of Hong Kong.

The Hong Kong healthcare system consists of public and private sectors. A major part of primary care is provided by the private sector, and the bulk of specialist and inpatient secondary and tertiary care are delivered through the public sector. All Hong Kong residents are eligible to receive care from the public sector at a heavily subsidized rate. Hospital fees account for only 2% of hospital revenue. The HA is the biggest public health service provider and provides more than 90% of total inpatient services in Hong Kong [10]. Patients with symptoms of COPD exacerbation normally go to the emergency department, and may in turn be admitted to the public hospital. In this study, we included all COPD admissions to public hospitals in Hong Kong from Jan 2006 to Dec 2007 in the analysis.

The objectives of this study were:

• To calculate admission and unplanned readmission rates of COPD patients in Hong Kong; and

• To identify potential factors contributing to unplanned readmission

## Methods

### Setting and subjects

Our dataset contained all the admission records to HA hospitals, from January 1 2006 to December 31 2007, which were captured from the HA computer system. To ensure confidentiality, patients' personal particulars such as names of patients and Hong Kong Identity Card numbers, were converted to dummy identities. The dataset was also protected by a password to ensure data security. This study was approved by the ethical committees of all service clusters of HA and was performed in accordance with the World Medical Association's Declaration of Helsinki.

Potential factors which might contribute to unplanned readmission were identified by literature review. However, the dataset did not contain all relevant variables. With the suggestion of the physician, we had determined to include acute length of stay (ALOS), Charlson comorbidity index, discharge status, age, gender, means of hospital payment and whether or not the patient lived in a nursing home in the analysis. We captured all patients' information with the principal diagnosis of COPD (ICD-9: 490-492, 494-496). The Charlson comorbidity index (CCI) was also computed to assess the interaction of comorbidity and COPD. The CCI was developed in 1987 based on one year mortality data from internal medicine patients [11]. The score encompasses 19 medical conditions weighted 1-6 with total score ranging from 0-37. It has been used to stratify patients in order to control for the confounding influence of comorbid conditions on overall survival [12]. It combines the risk from age and the risk from comorbid disease into a single variable, estimating the risk of death. A higher CCI indicates an increased severity of condition and a higher relative risk of death. CCI has been used to assess comorbidity of COPD patients [12-14]. The CCI was computed for each patient's admission to provide prognostic taxonomy for comorbid conditions.

The unplanned readmission status, ALOS and discharge status (deceased/home/convalescent bed/others) were also collected. There is no standardized definition of unplanned readmission. Previous studies which were based on statistical modelling such as survival analyses as well as the sensitivity and specificity analyses, had mathematically demonstrated that 30 days was an optimal choice for identifying unplanned readmission [15,16]. In this study we defined an unplanned readmission episode as an admission which followed a previous admission within a 30 day duration, excluding planned readmissions.

The dataset also provided demographic variables such as patients' age, gender, their hospital payment method and whether they were living in nursing homes. Patients receiving public assistance, such as disability allowance and Comprehensive Social Security Assistance (CSSA) Scheme for people with financial difficulties, would have their medical fees partly or totally waived.

### Data Analysis

Statistical analyses were performed using SPSS 16.0. Subjects' demographics were analyzed using descriptive statistics. Univariate analyses, including t-tests and chi-squared tests, were used to assess the association between unplanned readmission and independent variables including age, gender, receiving public assistance, living in nursing home and CCI. One-way ANOVA and pearson correlation were used to assess the associations between ALOS and independent variables including age, gender, receiving public assistance, living in nursing home, unplanned readmission and CCI. For multivariate analyses, logistic regression was used to assess potential factors which affected unplanned readmission of COPD subjects, and negative binomial regression test was used to identify potential determinants of ALOS. Negative binomial regression was used since ALOS had a highly skewed distribution with variance considerably greater than its mean. The identify link was used, meaning that ALOS was untransformed, in order to preserve ease of interpretation. A significance level of 0.05 was used in the analyses.

## Results

Among all the admission episodes retrieved from the HA computer system, 65497 (8.0%) episodes were identified with principal diagnosis of COPD in the year 2006 and 2007. Among these 65497 COPD admission episodes, 15882 (24.2%) were unplanned. Patients' demographics are shown in Table 1.

**Table 1:** Characteristics among 65497 COPD^ patient episodes

	N = 65497 n (%)
**Age (yrs), n = 65496**	
Mean ± SD	76.81 ± 9.59
**Gender**	
Male	50456 (77.0)
Female	15041 (23.0)
Payment*	
Public assistance	28693 (43.8)
Others	36804 (56.2)
**Living in nursing home**	
Yes	12576 (19.2)
No	52921 (80.8)
**Charlson comorbidity index**	
Mean ± SD	1.21 ± 0.74
**Unplanned readmission**	
Yes	15882 (24.2)
No	49615 (75.8)
**ALOS for COPD treatment**	
Mean ± SD	4.41 ± 7.53
**Death episode**	
Yes	1958 (3.0)
No	63523 (97.0)
missing	16

^ COPD: ICD-9-CM Diagnosis Code: 490-492, 494-496

*Public assistance: Patients eligible for complete or partial medical fee waiver

Table 2 shows the potential factors affecting unplanned COPD readmission episodes. It was found that patients with unplanned readmission episodes were slightly younger (76.74 ± 9.34 vs 76.83 ± 9.67, P = 0.020), more predominantly male (81.2% vs 75.7%, P < 0.001), more likely to be receiving public assistance (51.9% vs 41.2%, P < 0.001), and living in nursing homes (24.1% vs 17.6%, P < 0.001), compared with those who were not readmitted unplanned. However, CCI (1.20 ± 0.82 vs 1.21 ± 0.72, P = 0.053) was not significantly associated with the unplanned readmission episodes. In the multivariate analysis (Table 2), males (OR = 1.45, 95%CI 1.38-1.52), patients receiving public assistance (OR = 1.41, 95%CI 1.36-1.46) and living in nursing homes (OR = 1.41, 95%CI 1.34-1.47) were significantly more likely to have unplanned readmission episodes. It was also found that patients who were readmitted unplanned had a higher mortality rate than those who were not (3.4% vs 2.9%, P < 0.001).

**Table 2:** Analysis of potential factors affecting unplanned COPD^ patient readmission episodes

	Univariate Analysis			Multivariate Analysis (Logistic Regression)	
	**Unplanned Readmission**			**Unplanned Readmission**	
	**Yes (n = 15882)** **Mean ± SD**	**No (n = 49615)** **Mean ± SD**	**P**	**OR(95% CI)**	**P**

**Age (yrs)**	76.74 ± 9.34	76.83 ± 9.67	0.020	1.00(0.996-1.000)	0.049
**Gender**					
Male	12889 (81.2)	37567 (75.7)	< 0.001	1.45 (1.38-1.52)	< 0.001
Female	2993 (18.8)	12048 (24.3)		1.00 (reference)	
Public assistance*					
Yes	8235 (51.9)	20458 (41.2)	< 0.001	1.41(1.36-1.46)	< 0.001
No	7647 (48.1)	29157 (58.8)		1.00 (reference)	
**Living in nursing home**					
Yes	3820 (24.1)	8756 (17.6)	< 0.001	1.41 (1.34-1.47)	< 0.001
No	12062 (75.9)	40859 (82.4)		1.00 (reference)	
**Charlson comorbidity index**	1.20 ± 0.82	1.21 ± 0.72	0.053	0.99(0.96-1.01)	0.233

^ COPD: ICD-9-CM Diagnosis Code: 490-492, 494-496

*Public assistance: Patients eligible for complete or partial medical fee waiver

Table 3 shows the potential factors associated with ALOS in the univariate analyses. The negative binomial regression model results showed that female patients (β = 0.38, P < 0.0001), those who were readmitted unplanned (β = 0.39, P < 0.0001), with higher CCI (β = 0.32, P < 0.0001), receiving public assistance (β = 0.17, P = 0.0003) and living in nursing homes (β = 0.54, P < 0.001), had a longer ALOS compared with those who were not (Table 4). Note that β represents the mean difference between groups, i.e. those with unplanned readmission had a mean length of stay 0.39 day longer than those without unplanned readmission.

**Table 3:** Univariate Analysis of potential factors affecting ALOS for COPD^ treatment

	Univariate Analysis (ANOVA)	
	**ALOS** **Mean(d) ± SD**	**P**

**Age**	γ = 0.01	0.002
**Gender**		
Male	4.32 ± 7.40	< 0.001
Female	4.74 ± 7.93	
Public assistance*		
Yes	4.58 ± 7.68	< 0.001
No	4.28 ± 7.41	
**Living in nursing home**		
Yes	4.96 ± 7.59	< 0.001
No	4.28 ± 7.51	
**Unplanned readmission**		
Yes	4.71 ± 6.85	< 0.001
No	4.32 ± 7.73	
**Charlson comorbidity index**	γ = 0.03	< 0.001

^ COPD: ICD-9-CM Diagnosis Code: 490-492, 494-496

*Public assistance: Patients eligible for complete or partial medical fee waiver

γ = Pearson correlation

**Table 4:** Analysis of potential factors affecting ALOS for COPD^ treatment

	Negative Binomial Regression Model		
	**ß**	**Standard Error**	**P**

**Age**	0.0023	0.0024	0.3248
**Gender**			
Female	0.3803	0.0554	< 0.001
Male	0 (reference)		
Public assistance*			
Yes	0.1692	0.0464	0.0003
No	0 (reference)		
**Living in nursing home**			
Yes	0.5430	0.0652	< 0.001
No	0 (reference)		
**Unplanned readmission**			
Yes	0.3891	0.0533	< 0.001
No	0 (reference)		
**Charlson comorbidity index**	0.3171	0.0405	< 0.001

^ COPD: ICD-9-CM Diagnosis Code: 490-492, 494-496

*Public assistance: Patients eligible for complete or partial medical fee waiver

## Discussion

This is the first study reporting the characteristics of all COPD admissions to public hospital in Hong Kong. In our study, we found that there were high admission and unplanned readmission rates in Hong Kong and 8.0% of admission episodes to medical wards were COPD admissions. As medical admissions contributed to approximately half of the total admission in HA, our figure of 8.0% was comparable to the COPD annual admissions of 4% of the total admissions reported by HA in year 2008-2009 [17]. This figure was significantly higher than previously reported in UK, which was around 1% [4]. Admission and readmission to hospital are very common among COPD patients discharged from hospital after an acute exacerbation [18]. 80% of patients requiring inpatient management of a severe exacerbation are readmitted with a year [19]. Unplanned readmission has a great impact on hospital stay and healthcare expenses [20,21] but unplanned readmission of COPD is seldom reported. In our study, the unplanned readmission rate of COPD was 24.2%. Previous studies usually reported readmission of COPD only which varied from 11.6% (48 hour after discharge from the emergency room) to 63% (1 year after admission to a general hospital) [22].

This study showed that social factors were important in patients who were readmitted unplanned. Male patients, those who were receiving public assistance and those living in nursing homes were more likely to be readmitted unplanned. Studies have shown that patients who are poor or already receiving nursing care are more frequently hospitalized or readmitted unplanned [7,23-25]. Nursing home residents are usually frail. They often experience complications and suffer important functional and cognitive decline, and sometimes the medical deteriorations are unavoidable [26-28]. When patients are discharged, it is common that patients, caregivers and staff from nursing homes cannot get adequate clinical and social support from the community. They are not familiar with services in the community and lack of skills in managing COPD. Therefore, whenever the patients have problems, no matter it is really accounted for hospitalization; nursing home staff tend to send the residents to emergency department. Nursing home staff also tend to bring the residents to seek medical care more frequently and earlier even for minor exacerbations [25]. This explains the reasons of high hospital admission and unplanned readmission rates, and those who received public assistance were much more likely to have unplanned readmission. Patients who receive public assistance are those with financial hardship. Their economic situation may affect the possibilities of obtaining home health assistance, keeping scheduled home care programmes or obtaining adequate home equipment [24]. Further studies are necessary to investigate the reasons of higher risk of unplanned readmission in male patients and whether smoking status is a potential factor in these patients.

Unplanned readmission, higher CCI, patients who were receiving public assistance and living in nursing home were found to be factors contributing to longer ALOS after adjusted with other covariates. COPD patients who were readmitted unplanned stayed 0.39 days longer in acute ward than those who were not readmitted unplanned. Conditions of these patients could be more complicated or severe. They might allow to be discharged only when their conditions were stable and appropriate examinations and interventions had been completed, therefore, patients who were readmitted unplanned could have a longer acute length of stay. Of those patients who had a high CCI, it was understandable that they had poor pre-morbid status as well as multiple comorbidities, therefore, they were likely to stay longer. Arrangement of social services was also necessary for some patients who lived in nursing homes and supported by public assistance before they could be discharged.

The Hong Kong healthcare system is comparable to those of many western developed countries. Hong Kong has a low infant mortality rate and maternal mortality rate, and one of the longest life expectancies for both sexes in the world [29]. The healthcare professionals and treatments are up to international standard. However, our study showed that more than one fifth of COPD patients were readmitted unplanned. It might due to a fragmented care pathway and lack of continuity of care of COPD patients. The Hong Kong healthcare system is a dual system but both the private and public sectors are running independently. The majority of the COPD patients in Hong Kong are regularly followed up in public-operated clinics. When patients show symptoms of exacerbation or have problems after discharge, they may not be suffering from severe conditions or poor pre-morbid states. A number of strategies have been shown to reduce incidence of exacerbation effectively, including smoking cessation programmes, hospital at home service, and self-management [30]. These programmes are also available in Hong Kong but their operation is far from systematic [31]. Other programmes such as "Community Call Centre", patients receiving follow-up phone calls from nurses within 48 hours post-discharge; and "Day Centre", patients receiving treatments in the community, are also provided but only in a few public hospitals. Because of the possibility of ineffectiveness of resource allocation and the fragmentation of healthcare delivery system, only a limited proportion of COPD patients can receive these services. As a result, patients who require management of exacerbation would have a higher chance to be readmitted. Those who can afford private healthcare services may go to the private sector, while those who are not able to afford would seek help from the Emergency Department and admitted back to public hospitals.

High hospitalization rates can lead to increase in healthcare costs. Since patients readmitted unplanned usually stay longer in hospitals, if we could reduce the unplanned readmission rate, the cost saving would be more significant. A good discharge planning process should be developed within the system so that the care pathway would be connected from the hospital to the community. An early discharge planning process should be introduced as soon as patients are admitted. The process should involve checklist to identify patients who are likely to be readmitted, start the pulmonary rehabilitation programme early, and coordinate team to follow up when patients are discharged home. This could in turn improve the efficiency of health service delivery to COPD patients, including both in-hospital and community, and reduce the high cost of hospitalization and re-hospitalization.

## Limitations

Our study assessed retrospectively the frequency of and risk factors for hospital admission and unplanned readmission in COPD patients. Although data from private hospitals were not available, they were not expected to have any significant influence on the results, as according to the data in 1998, only 6.8% of total hospital days were being utilized in private hospitals [25]. As HA took up more than 90% of all hospital admissions [32], the findings of this study could be taken to represent the real-life situation in Hong Kong population. Although clinical data on the index admission and patients' high risk factors of COPD were not available in the dataset obtained, CCI was computed to assess comorbidity of the patients. Smoking status which is a potential confounding factor was also not available in the dataset. Further studies are necessary to investigate its impact on the risk of unplanned readmission of the male patients.

## Conclusions

Unplanned readmission of COPD patients has a huge impact on the public healthcare system. Our study has identified several important factors, including social factors, within the local context. A systematic approach in programme provision in the community and a good discharge planning process targeting on patients who are at high risk of unplanned readmission are essential.

## Competing interests

The authors declare that they have no competing interests.

## Authors' contributions

FWC and FYW drafted the manuscript, performed data analysis and interpreted the data and results. WBG performed data analysis and interpreted the data and results. EKY performed manuscript editing. All authors were involved in the concept and designing the study and approved the final manuscript.

## Pre-publication history

The pre-publication history for this paper can be accessed here:

http://www.biomedcentral.com/1472-6963/11/186/prepub
